# Core self-evaluation and school disengagement in early adolescence: a three-wave longitudinal network analysis

**DOI:** 10.3389/fpsyg.2026.1809883

**Published:** 2026-07-03

**Authors:** Jing Liu, Haiyan Liu, Haiqi Zhang

**Affiliations:** 1 School of Psychology, Hainan Normal University, Haikou, China; 2 Adolescent Psychological Development and Education Center of Hainan, Haikou, China

**Keywords:** core self-evaluation, early adolescents, longitudinal study, network analysis, protective factors, school disengagement

## Abstract

School disengagement during early adolescence is a persistent concern associated with a range of adverse academic and psychological outcomes. Although core self-evaluations (CSE) have been identified as an important protective factor, prior research has mainly relied on variable-centered approaches and provides limited insight into fine-grained associations and their temporal dynamics. To address this gap, the present study adopted a three-wave longitudinal design among junior high school students in China (*N* = 896) and applied psychological network analysis to examine item-level associations between CSE and school disengagement across three time points. Network models were estimated separately at each wave, and network comparison tests were conducted to examine structural and global connectivity changes over time. Results indicated that the overall network structure remained largely stable across the three waves. CSE items were consistently negatively associated with cognitive, behavioral, and emotional disengagement, while disengagement components showed strong within-domain associations across time points. Cognitive disengagement consistently emerged as a central node within the networks, whereas emotional disengagement showed increased centrality at later waves. Longitudinal comparisons further suggested only modest changes in global connectivity and node-level influence. Overall, the findings suggest that CSE and school disengagement are characterized by relatively stable patterns of associations across early adolescence, extending previous research by providing a fine-grained network perspective on their interrelations and offering implications for targeting key disengagement components and supporting self-evaluative resources in interventions.

## 1 Introduction

Early adolescence is a critical developmental period characterized by increasing academic demands, heightened psychological load, and rapid changes in self-awareness. During this stage, school disengagement (SD) has emerged as an important indicator of maladaptive learning experiences. It reflects a multidimensional pattern of reduced cognitive investment, emotional exhaustion, and diminished behavioral participation in academic activities, and has been consistently linked to negative outcomes such as declining academic performance, reduced classroom engagement, and increased mental health risks (Schaufeli et al., 2002; Salmela-Aro and Upadyaya, 2014). Longitudinal research further indicates that school disengagement tends to exhibit relatively stable patterns across junior high school years (Wang and Eccles, 2012; Ding et al., 2022; Gao, 2023; Zhang et al., 2025). Moreover, disengagement has been associated with a range of psychological and contextual risk factors, including depression, anxiety, academic stress, impaired self-concept, and reduced social support (Salmela-Aro and Upadyaya, 2014; Martinot et al., 2020; Zhao et al., 2023). Given its developmental persistence and potential negative consequences, understanding the factors associated with school disengagement is of considerable importance for both theory and educational practice.

Among the various individual factors influencing school disengagement, core self-evaluation (CSE) has received increasing attention as a higher-order personality construct. CSE integrates four fundamental components—self-esteem, self-efficacy, emotional stability, and locus of control—and reflects individuals’ overall evaluation of their own worth and capabilities (Judge et al., 2003). From a theoretical perspective, the Conservation of Resources theory (Hobfoll, 1989) suggests that individuals rely on stable internal psychological resources to cope with stress and manage demands. As a core personal construct, CSE is associated with how individuals appraise academic challenges and relate to cognitive, emotional, and behavioral aspects of their academic experiences. Previous research has shown that higher levels of CSE are associated with stronger self-regulation, greater resilience, and more adaptive coping strategies in response to stress (Chang et al., 2012; Kammeyer-Mueller et al., 2009; Yang et al., 2021). In educational contexts, CSE has been linked to lower levels of academic stress and burnout, as well as fewer emotional exhaustion and avoidance behaviors (Seçer and Ulaş, 2020; Li et al., 2024). Conversely, lower CSE has been associated with maladaptive attribution patterns, feelings of helplessness, and higher likelihood of cognitive, emotional, and behavioral disengagement under sustained academic pressure (Ye et al., 2025; Li et al., 2024). Taken together, these findings suggest that CSE is meaningfully associated with school disengagement and may relate to individual differences in stress appraisal, psychological resources, and adaptation to academic demands.

Despite this growing body of research, several limitations remain. First, most existing studies have primarily relied on total scores or latent variable approaches, which may obscure heterogeneous associations among specific psychological components. Second, school disengagement is inherently multidimensional, encompassing cognitive, behavioral, and emotional aspects that may interact in complex and potentially asymmetric ways, yet these interactions are rarely examined at a fine-grained level. Third, although some longitudinal evidence exists, relatively few studies have examined how the associations between core self-evaluation and different dimensions of school disengagement may vary across time points. As a result, the micro-level structural associations and potential changes over time remain insufficiently characterized.

Addressing these gaps is essential for advancing theoretical understanding of adolescent academic adaptation and for informing more targeted intervention strategies. In particular, a more fine-grained examination of the associations between core self-evaluation and school disengagement, along with potential changes across time, may provide deeper insights into the structural patterns linking these constructs.

### 1.1 Risk–protective perspective

From a risk–protective framework and resource-regulation perspective, students’ learning attitudes and behaviors can be understood as the result of interactions between internal psychological resources (protective factors) and various stressors (risk factors). Within this framework, core self-evaluations (CSE) can be conceptualized as a fundamental internal resource associated with how individuals perceive, interpret, and respond to academic challenges. Specifically, individuals with higher levels of CSE tend to report stronger self-efficacy, greater emotional stability, and a higher sense of control, which have been linked to more effective emotion regulation and coping strategies when facing academic stress (Lian et al., 2014; Leupold et al., 2020; Paloș, 2024). These resource characteristics are associated with maintaining a sense of meaning in learning, sustaining behavioral engagement, and experiencing fewer negative emotional states, which in turn are related to lower levels of school disengagement (Putwain et al., 2024; Ragusa et al., 2023).

Conversely, school disengagement and academic burnout have been associated with multiple interacting risk factors, including maladaptive learning cognitions, persistent negative emotions, and behavioral withdrawal. Prior research suggests that cognitive perceptions such as “perceived meaninglessness of learning” are closely linked to emotional exhaustion and disengaged behaviors, reflecting the interconnected nature of cognitive, emotional, and behavioral processes (Zhang et al., 2025; Zhao et al., 2024). Negative emotional states such as depression and anxiety have also been found to correlate with higher levels of disengagement and reduced motivational and psychological resources (Zhang et al., 2025; Qin et al., 2025). Individuals with lower levels of CSE tend to report lower perceived control and coping capacity, which is associated with higher levels of emotional exhaustion and behavioral withdrawal (Lian et al., 2014; Leupold et al., 2020).

Taken together, this perspective provides a theoretical foundation for understanding the associations between CSE and school disengagement, suggesting that higher CSE is meaningfully related to lower disengagement through differences in stress appraisal, psychological resources, and adaptation to academic demands. Rather than reflecting a simple linear association, these relationships likely involve complex interrelations among cognitive, emotional, and behavioral components, highlighting the importance of examining them within a systems framework.

### 1.2 Network analysis

Network analysis has emerged as a methodological approach in psychology for examining complex multivariate systems, providing a framework to investigate direct associations among observed variables such as items of core self-evaluations (CSE) and school disengagement. Unlike traditional latent variable models that assume observed variables reflect underlying constructs, network analysis conceptualizes psychological phenomena as systems of interacting components, where nodes represent variables and edges indicate conditional associations after controlling for all other variables in the network. This approach allows for the identification of fine-grained interrelations that may be obscured in aggregate-level analyses (Epskamp et al., 2018). A key advantage of network analysis is its ability to identify central and bridge nodes within psychological systems. Central nodes are typically highly connected and tend to show stronger associations with other components, whereas bridge nodes can link distinct domains such as cognitive, emotional, and behavioral aspects of school disengagement. Prior research has shown that certain cognitive components (e.g., perceived meaninglessness of learning) tend to occupy central positions and are associated with both academic stress and emotional or behavioral disengagement, while self-efficacy–related components tend to be negatively associated with disengagement indicators within the network (Mulder and Hamaker, 2021; Robinaugh et al., 2016; Zhang et al., 2025; Paloș, 2024).

Psychological networks may vary across time points. Repeated network estimation across multiple time points allows researchers to compare network structures across measurement waves, examining similarities and differences in node centrality, edge strength, and overall connectivity across time points (Bringmann et al., 2015; Epskamp et al., 2018). This approach provides a more detailed understanding of temporal patterns in psychological associations, complementing traditional longitudinal approaches that primarily focus on mean-level change. Overall, network analysis provides a framework for examining the structural organization of psychological systems and comparing association patterns across time points, thereby facilitating a more nuanced understanding of the relationships between core self-evaluations and school disengagement during adolescence.

### 1.3 The current study

The relationship between core self-evaluations (CSE) and school disengagement in early adolescence is multidimensional and complex, yet existing research has paid limited attention to the item-level structural associations between these constructs and their differences across time points. To address this gap, the present study employed a three-wave longitudinal design, collecting data on CSE (10 items) and the three dimensions of school disengagement—cognitive (7 items), behavioral (6 items), and emotional (4 items)—at three time points (December 2024, April 2025, and September 2025).

The primary objectives of this study were to: (1) construct cross-sectional psychological networks of CSE and school disengagement at each time point; (2) compare network structures across time using network comparison analysis; and (3) identify central and bridge nodes within and between the CSE and school disengagement networks at the item level.

## 2 Methods

### 2.1 Participants

This study employed a convenience sampling approach, recruiting students from three junior high schools in Haikou, Hainan Province, China. All participants were required to understand and independently complete the questionnaires. Prior to data collection, informed consent was obtained from the schools, parents, and the students themselves. The study protocol was approved by the Ethics Committee of the School of Psychology at Hainan Normal University and adhered to the ethical guidelines outlined in the Declaration of Helsinki for research involving minors. Data were collected at three time points: December 2024 (T1), April 2025 (T2), and September 2025 (T3). A total of 1,046 students participated at baseline, of whom 896 completed all three waves and were included in the final analysis, yielding a retention rate of 85.66% and a dropout rate of 14.34%. Participants were excluded if they failed to complete one or more waves or showed substantial missing data or inattentive response patterns (e.g., identical responses across items).

To examine potential attrition bias, independent-samples t-tests were conducted comparing retained participants (*n* = 896) and excluded participants (*n* = 150) on baseline measures. For core self-evaluations (CSE), retained participants showed slightly higher scores (*M* = 32.98, SD = 7.05) than excluded participants (*M* = 31.38, SD = 6.91), *t*_1044_ = 2.58, *p* = 0.01, Cohen’s *d* = 0.23, 95% CI (0.06, 0.40). For school disengagement, no significant differences were found between retained participants (*M* = 40.38, SD = 14.82) and excluded participants (*M* = 41.91, SD = 13.68), *t*_1044_ = −1.19, *p* = 0.24, nor for its cognitive (*t*_1044_ = −1.07, *p* = 0.29), behavioral (*t*_1044_ = −0.74, *p* = 0.46), and emotional dimensions (*t*_1044_ = −1.51, *p* = 0.13). Although a small but statistically significant difference was observed for CSE, the effect size was small, and no differences were found for school disengagement or its subdimensions, indicating that attrition is unlikely to have substantially affected the observed associations in the primary analyses. The final sample included 472 boys and 424 girls aged 12–14 years (*M* = 12.95, SD = 0.71), and additional demographic characteristics are reported in Table 1.

**Table 1:** Demographic characteristics of the participants.

Variable	Category	*n* (%)	Variable	Category	*n* (%)
Gender	Boys	472 (52.68)	Family structure	Intact family	826 (92.19)
	Girls	424 (47.32)		Divorced family	47 (5.25)
Grade	7	491 (54.80)		Reconstituted family	15 (1.67)
	8	405 (45.20)		One parent deceased	8 (0.89)
Residence	Urban	738 (82.37)	Father’s education	Junior high or below	186 (20.76)
	Rural	158 (17.63)		High school/technical school	257 (28.68)
Left-behind status	Yes	36 (4.02)		College or above	453 (50.56)
	No	860 (95.99)	Mother’s education	Junior high or below	238 (26.56)
Only-child status	Yes	155 (17.30)		High school/technical school	266 (29.69)
	No	741(82.70)		College or above	392 (43.75)

### 2.2 Measures

Standardized questionnaires were used to assess the two core constructs of this study—core self-evaluations (CSE) and school disengagement—at all three time points. All measures were administered in Chinese and have demonstrated good reliability and validity. Internal consistency coefficients (Cronbach’s *α*) for the three waves in this study were satisfactory, with specific reliability indices presented in Table 2. Longitudinal measurement invariance across the three waves was further examined using confirmatory factor analysis (CFA) in the lavaan package in R. The configural, metric, and scalar invariance models demonstrated acceptable fit indices (CFI > 0.90, RMSEA < 0.08), and changes in comparative fit index remained below the recommended cutoff (ΔCFI < 0.01), indicating that longitudinal measurement invariance was supported across the three measurement occasions (Supplementary Table S1). CSE, as a higher-order personality construct, reflects an individual’s overall evaluation of their own abilities and self-worth and is associated with adolescents’ cognitive, emotional, and behavioral adaptation in academic contexts. This construct originates from the theoretical framework proposed by Judge et al. and has been widely applied in adolescent research (Paloș, 2024). School disengagement, encompassing cognitive, behavioral, and emotional dimensions, represents a maladaptive learning pattern closely related to psychological distress and has been increasingly included in studies of academic burnout and learning adaptation in recent years (Gao, 2023; Li et al., 2024).

**Table 2:** The information of the questionnaires.

Measurement	Questionnaire	Variable	Items	Cronbach’s α		
				T1	T2	T3
Protective factors	Core self-evaluation scale	CSE	10	0.84	0.83	0.81
Risk factors	School disengagement scale	Cognitive dimension	7	0.90	0.89	0.86
		Behavioral dimension	6	0.87	0.84	0.78
		Emotional dimension	4	0.91	0.86	0.82

Time1 = 2024.12, Time2 = 2025.4, Time3 = 2025.9, total sample = 896.

#### 2.2.1 Core self-evaluations

CSE was measured using the Core Self-Evaluations Scale (CSES) developed by Judge et al. (2003), adapted to the Chinese context based on local validation and revision studies (Wei et al., 2013). The final version employed in this study is a simplified Chinese adaptation suitable for the current sample (Appendix A). The scale assesses four dimensions—self-esteem, self-efficacy, emotional stability, and locus of control—to reflect individuals’ overall evaluation of their personal value, with higher scores indicating higher CSE (Judge et al., 2003). The Chinese version has demonstrated good internal consistency and construct validity in Chinese samples and is widely used for measuring adolescents’ CSE in China (Gao, 2023; Paloș, 2024). Items were rated on a 5-point Likert scale (1 = strongly disagree, 5 = strongly agree). Cronbach’s *α* for the three waves in this study ranged from good to excellent (Table 2), consistent with reliability levels reported for adolescent populations (Paloș, 2024).

#### 2.2.2 School disengagement

School disengagement was assessed using the School Disengagement Scale for Junior High School Students developed by Zhao (2019) (Appendix B). The scale includes 17 items across three dimensions: cognitive disengagement (7 items), behavioral disengagement (6 items), and emotional disengagement (4 items). The cognitive dimension evaluates students’ negative perceptions of learning significance and value, the behavioral dimension reflects withdrawal and fatigue in learning activities, and the emotional dimension assesses emotional exhaustion and negative affect experienced in learning contexts. Items are rated on a 5-point Likert scale (1 = strongly disagree, 5 = strongly agree), with higher scores indicating higher levels of disengagement. Zhao (2019) demonstrated that the scale possesses good structural validity and reliability, effectively capturing adolescents’ cognitive, behavioral, and emotional aspects of school disengagement. In this study, Cronbach’s α for all three waves was in the moderate-to-high range (Table 2), indicating reliable internal consistency and measurement stability. This scale is suitable for examining the associations between CSE and school disengagement in junior high students (Gallé-Tessonneau and Gana, 2019; Paloș, 2024; Putwain et al., 2024).

### 2.3 Statistical analysis

Descriptive statistics were conducted using IBM SPSS 27.0, while all network analyses were performed in R (Version 4.5.2). The main R packages used in the analyses included bootnet (version 1.8), qgraph (version 1.9.8), NetworkComparisonTest (version 2.2.3), and lavaan (version 0.6.20). The analytical procedures included network estimation, centrality assessment, network accuracy and stability testing, and network comparison. These methods allow for the identification of direct relationships between individual items of core self-evaluations (CSE) and school disengagement and enable the examination of similarities and differences in network structures across time points. Such procedures have become standard in contemporary psychological network research (Borsboom and Cramer, 2013; Epskamp et al., 2018; van Borkulo et al., 2023).

#### 2.3.1 Network estimation

The EBICglasso method implemented in the “bootnet” and “qgraph” packages in R (Version 4.5.2) was used to estimate and visualize the network structures (Epskamp et al., 2012; Epskamp et al., 2018). The EBIC hyperparameter *γ* was set to 0.5, as recommended, to obtain a more parsimonious and interpretable network structure (Epskamp et al., 2018), while the tuning parameter *λ* was selected automatically by the estimation procedure. Since the variables in the present study were measured using Likert-type scales, Spearman correlations were computed using the corMethod = “spearman” setting in the estimateNetwork() function to generate the partial correlation matrices. Missing data were handled using listwise deletion prior to network estimation.

In the estimated networks, nodes represent individual items of core self-evaluations (CSE) and school disengagement (SD), and edges represent regularized partial correlations between two nodes after controlling for all other nodes in the network. Positive associations are displayed as solid red edges, while negative associations are shown as dashed green edges. Edge thickness reflects the magnitude of associations, referred to as edge weights, with partial correlation coefficients ranging from −1 to 1 (Haslbeck and Fried, 2017).

#### 2.3.2 Centrality estimation

To identify key nodes, two centrality indices were calculated: node strength and expected influence (EI). Node strength reflects the sum of absolute edge weights connected to a node and is a classic measure of node connectivity within the network (Bringmann et al., 2019). EI accounts for the sign of edges, providing a more accurate representation of a node’s overall associations in networks containing both positive and negative associations (Robinaugh et al., 2016). Given that the network included two primary constructs (CSE and school disengagement), bridge centrality was also computed to identify nodes that show stronger associations across different psychological domains. Prior studies have reported the relevance of such nodes in connecting multiple constructs within multidimensional psychological networks (Jones et al., 2021). Centrality indices were computed based on the estimated weighted networks obtained from the EBICglasso procedure.

#### 2.3.3 Accuracy and stability estimation

To evaluate the accuracy and stability of the estimated networks, bootstrap procedures were conducted using the bootnet package (Epskamp et al., 2018). First, nonparametric bootstrapping with 1,000 iterations was used to estimate 95% confidence intervals (CIs) for edge weights, with narrower CIs indicating greater estimation accuracy. Bootstrap difference tests were performed to examine differences in centrality indices across nodes, including node strength and expected influence.

Case-dropping subset bootstrap procedures were then conducted to assess the stability of centrality estimates, including strength, expected influence, bridge strength, and bridge expected influence. Centrality stability coefficients (CS-coefficients) were calculated to quantify the robustness of these indices. According to recommended guidelines, a CS-coefficient above 0.25 indicates acceptable stability, whereas values above 0.50 indicate good stability for centrality estimation (Epskamp et al., 2018).

#### 2.3.4 Network comparison

To examine structural differences across the three time points, the Network Comparison Test (NCT) implemented in the Network Comparison Test package in R was conducted (van Borkulo et al., 2023). This permutation-based procedure was used to compare overall network structure (network invariance), global strength, and edge weight differences between networks across time points. Holm correction was applied for multiple comparisons in the edge-level analyses.

## 3 Results

### 3.1 Descriptive and statistical analysis

Table 3 presents the means, standard deviations, repeated-measures ANOVA results, effect sizes (*η*^2^_p_), 95% confidence intervals, and *post-hoc* pairwise comparisons for core self-evaluations (CSE) and the three dimensions of school disengagement (cognitive, behavioral, and emotional) across T1, T2, and T3. Overall, significant differences across time points were observed for all variables. Specifically, CSE scores differed significantly across the three waves (*F*(1.72, 1537.95) = 97.64, *p* < 0.001, *η*^2^_p_ = 0.10). *Post-hoc* pairwise comparisons with Bonferroni correction indicated significant differences between T1 and T2 and between T2 and T3, whereas the difference between T1 and T3 was not significant. These findings are generally consistent with previous research suggesting that adolescents’ self-concept may vary across academic and developmental contexts (Postigo et al., 2022; Rodriguez Buritica et al., 2025).

**Table 3:** Descriptive statistics, repeated measures ANOVA results, and *post-hoc* comparisons across T1, T2, and T3.

Variable	Mean (SD)			*F* (df)	*η* ^2^_p_	95% CI			*Post-hoc* comparisons (MD) (95% CI)		
	T1	T2	T3			T1	T2	T3	T1 vs. T2	T2 vs. T3	T1 vs. T3
CSE	32.98 (7.05)	29.64 (9.08)	32.90 (6.35)	97.64*** (1.72, 1537.95)	0.10	32.5, 33.5	29.0, 30.2	32.5, 33.3	3.34*** (2.63, 4.06)	−3.26*** (−3.97, −2.54)	0.09 (−0.42, 0.59)
Cognitive	15.71 (6.24)	16.52 (5.78)	16.86 (5.31)	19.44*** (1.98, 1770.60)	0.02	15.3, 16.1	16.2, 16.9	16.5, 17.2	−0.81*** (−1.25, −0.37)	−0.33 (−0.78, 0.11)	−1.15*** (−1.62, −0.67)
Behavioral	14.67 (5.68)	15.18 (5.03)	15.52 (4.31)	11.87*** (1.96, 1750.50)	0.01	14.3, 15.1	14.9, 15.5	15.2, 15.8	−0.51* (−0.94, −0.08)	−0.34 (−0.73, 0.05)	−0.85*** (−1.29, −0.41)
Emotional	9.99 (4.46)	10.18 (3.80)	10.48 (3.41)	6.57** (1.96, 1757.47)	0.01	9.7, 10.3	9.9,10.4	10.3,10.7	−0.18 (−0.51, 0.15)	−0.31* (−0.61, 0.00)	−0.49** (−0.83, −0.14)

CSE, core self-evaluation; SD, standard deviation; MD, mean difference; CI = 95% confidence interval; *η*^2^_p_ = partial eta squared. *** *p* <0.001, ** *p* <0.01, * *p* <0.05. *Post-hoc* comparisons were conducted following repeated measures ANOVA with Bonferroni correction for multiple comparisons. The sphericity assumption was violated. *F* values are reported with Greenhouse–Geisser correction.

Significant differences across time points were observed for all three dimensions of school disengagement. Cognitive disengagement scores differed significantly across the three waves (*F*(1.98, 1770.60) = 19.44, *p* < 0.001, *η*^2^_p_ = 0.02). *Post-hoc* pairwise comparisons with Bonferroni correction indicated significant differences between T1 and T2 and between T1 and T3, while the difference between T2 and T3 was not significant. Behavioral disengagement also differed significantly across time (*F*(1.96, 1750.50) = 11.87, p < 0.001, *η*^2^_p_ = 0.01), with *post-hoc* comparisons showing significant differences between T1 and T2 and between T1 and T3, whereas T2 vs. T3 was not significant. Emotional disengagement also showed significant changes over time (*F*(1.96, 1757.47) = 6.57, *p* < 0.01, *η*^2^_p_ = 0.01), with significant differences between T2 and T3 and between T1 and T3, but no significant difference between T1 and T2. These findings align with previous research linking academic pressure, school burnout, and school avoidance among adolescents (Simoës-Perlant et al., 2022; Ulaş and Seçer, 2024). Overall, the descriptive and statistical results indicate that CSE and school disengagement scores differed across time points, providing a robust basis for the subsequent network analyses.

### 3.2 Network estimation

**Figure 1:**
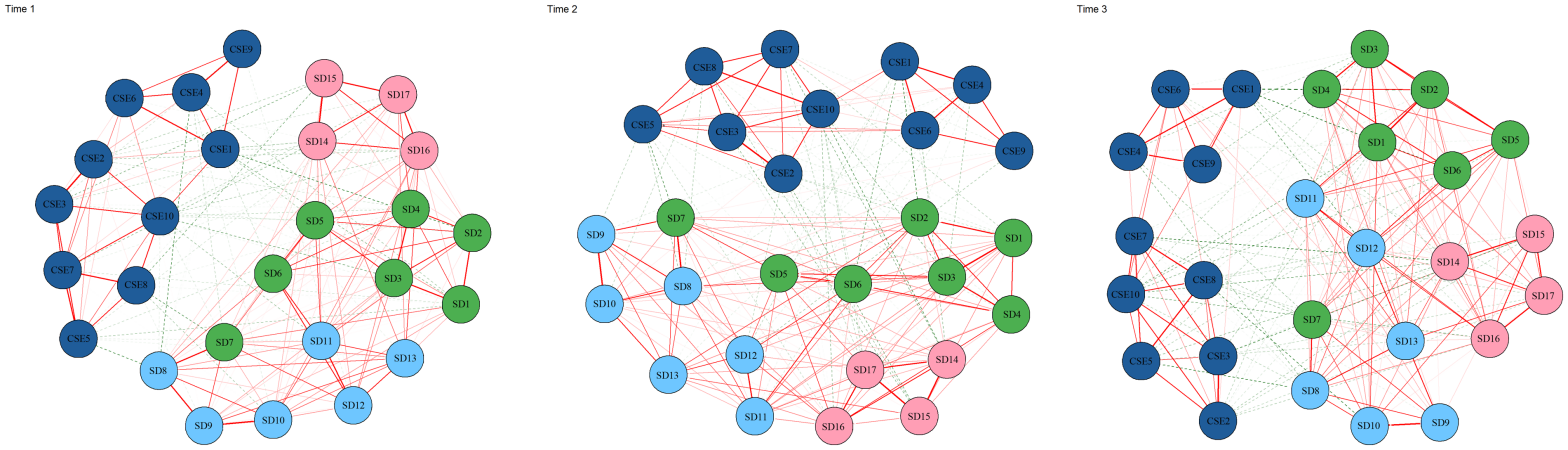
The network of all variables (*N* = 896). Core Self-Evaluation (dark blue nodes, CSE1- CSE10), School Disengagement (Cognition: green nodes, SD1–SD7; Behavior: light blue nodes, SD8–SD13; Emotion: pink nodes, SD14–SD17). Red lines are positive connections, dashed green lines are negative connections. Time1 = 2024.12, Time2 = 2025.4, Time3 = 2025.9.

Figure 1 illustrates the psychological network structures of Core Self-Evaluations (CSE) and the three dimensions of school disengagement (SD)—cognitive, behavioral, and emotional—across T1, T2, and T3, encompassing 27 nodes and 351 possible edges (calculated as 27*(27–1)/2). Among these, 181 edges had nonzero weights (mean weight = 0.03) at T1, 180 edges (mean weight = 0.03) at T2, and 191 edges (mean weight = 0.03) at T3. Overall, the networks across the three time points showed relatively consistent clustering patterns, with CSE items tending to cluster on one side and school disengagement items clustering on the other. This pattern indicates that items within the same construct were more strongly associated with each other, whereas associations between constructs were generally weaker. Across the three networks, the strongest positive edge weights were consistently observed between SD9 and SD10 (T1/T2/T3 = 0.37/0.34/0.35), reflecting close associations between poor study habits and lack of study planning. Strong positive associations were also identified between SD14 and SD15 at T1 and T2 (0.36/0.31), between CSE2 and CSE3 at all three time points (0.35/0.33/0.35), and between CSE7 and CSE8 at T1 (0.30). At T3, additional strong positive edges emerged between SD1 and SD2 (0.32) and between SD3 and SD4 (0.32), suggesting relatively strong associations among cognitive disengagement items. These findings indicate that both CSE items and disengagement items showed substantial within-construct associations across time points.

Across the networks, CSE items generally showed negative associations with school disengagement items, suggesting that higher CSE scores were associated with lower levels of cognitive, behavioral, and emotional disengagement. The strongest negative associations were observed between CSE5 and SD8 at T1 (−0.21) and between CSE1 and SD2 at T3 (−0.22), indicating inverse relationships between positive self-evaluations and disengagement-related learning attitudes and behaviors. These negative associations were distributed across multiple CSE and disengagement items rather than being restricted to isolated nodes, reflecting broad inverse relationships at the network level (Sun et al., 2025; Yudes et al., 2025). Previous research has similarly reported negative correlations between CSE and academic burnout, indicating that positive self-evaluation is associated with lower reported emotional exhaustion and behavioral withdrawal (Zhang et al., 2025; Gao, 2023). Within the disengagement items, positive associations were observed among cognitive, behavioral, and emotional nodes at all time points, with cognitive disengagement items often associated with both behavioral and emotional components. Positive associations between behavioral and emotional disengagement items appeared relatively stronger at T2 and T3, reflecting greater co-occurrence of these aspects during these time points (Gao, 2023; Iuga et al., 2025). Although edge weights and local layouts varied across time points, the overall pattern of associations remained relatively consistent across the three waves. These observations provide a descriptive account of item-level associations and form the basis for subsequent network comparison analyses (Supplementary Table S2).

### 3.3 Network centrality

**Figure 2:**
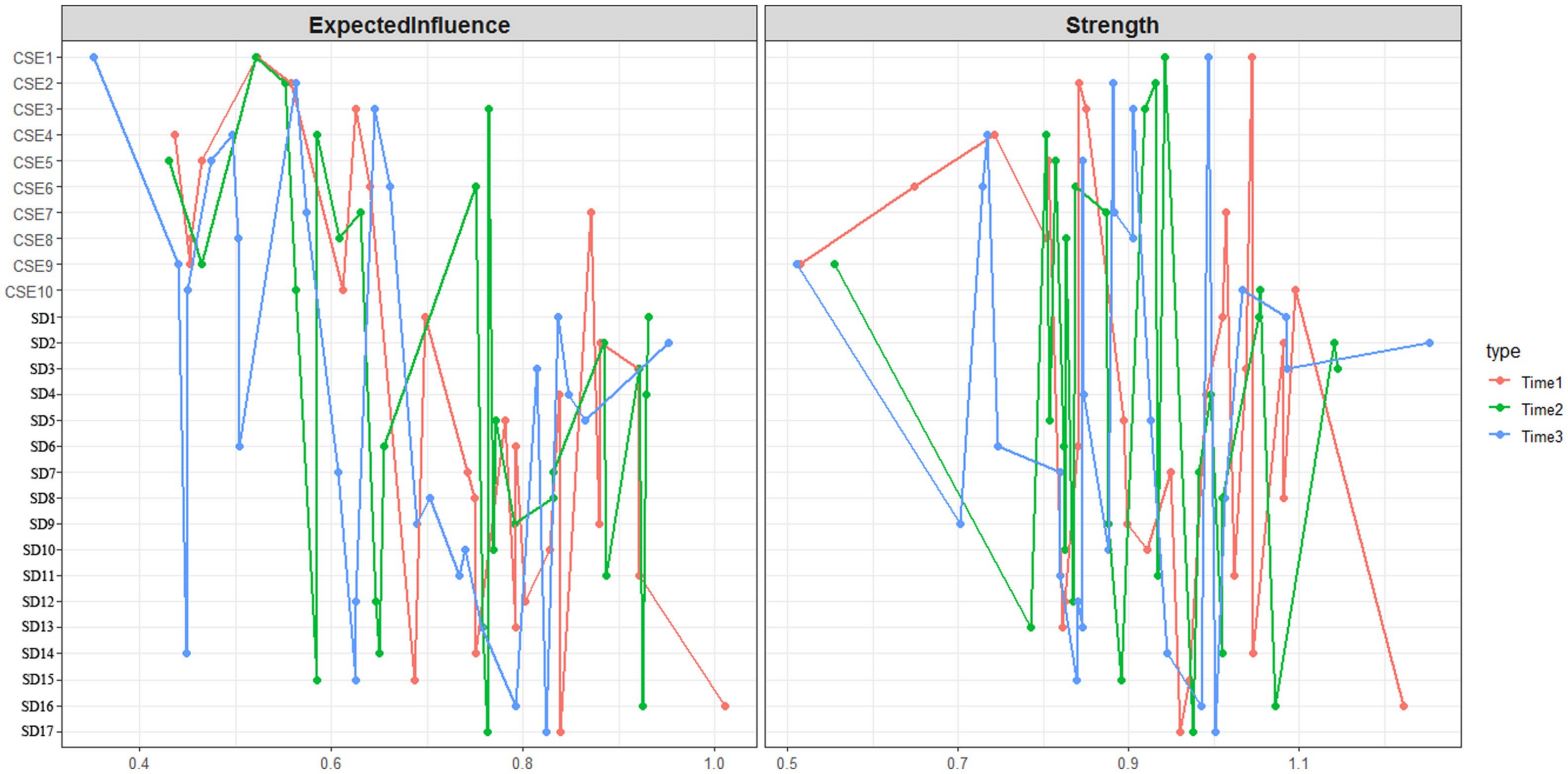
Centrality plots for the nodes depicted as Strength, Expected Influence. The X-axis represents standardized z-scores of these three centrality indices (the higher the value, the more central the node), and the Y-axis represents the variables. Time1 = 2024.12, Time2 = 2025.4, Time3 = 2025.9, total sample = 896.

Figure 2 presents the centrality indices of nodes in the core self-evaluations (CSE) and school disengagement (SD) networks across T1, T2, and T3. At T1, SD16 showed the highest strength value (2.05), followed by SD8 (1.19) and CSE10 (1.12). At T2, SD3 exhibited the highest strength (1.74), followed by SD2 (1.65) and SD16 (1.35). At T3, SD2 showed the highest strength value (2.68), followed by SD1 (1.24) and SD3 (1.19) (Supplementary Table S3). These findings indicate that several cognitive, behavioral, and emotional disengagement items consistently occupied relatively central positions within the network structure across the three time points. In particular, cognitive disengagement items (e.g., SD1–SD3) and emotional disengagement items (e.g., SD16) demonstrated relatively high connectivity with other nodes, suggesting that these nodes maintained relatively strong associations within the overall disengagement network.

Expected influence (EI) indices further revealed differences in positive and negative associations across nodes. At T1, SD16 showed the highest positive EI value (1.73), followed by SD11 (1.37) and CSE7 (1.21). In contrast, CSE5 (−1.78), CSE9 (−1.73), and CSE8 (−1.68) showed the largest negative EI values. At T2, SD1 (1.55), SD3 (1.51), and SD16 (1.35) exhibited the highest positive EI values, whereas CSE5 (−1.96), CSE9 (−1.57), and CSE2 (−1.22) showed the strongest negative EI values. By T3, SD2 demonstrated the highest positive EI value (2.08), followed by SD3 (1.18) and SD1 (1.06), while CSE1 (−2.14), SD14 (−1.32), and CSE9 (−1.25) showed the largest negative EI values (Supplementary Table S3). Overall, cognitive disengagement items consistently demonstrated relatively high positive expected influence across time points, whereas several CSE items consistently exhibited relatively large negative expected influence values. This pattern suggests that disengagement-related cognitive and emotional experiences were strongly interconnected within the network, while CSE items tended to show inverse associations with disengagement-related nodes. These findings are generally consistent with previous network studies reporting close associations between negative academic experiences and disengagement-related emotional and behavioral responses (Zhang et al., 2025; Gao, 2023; Yudes et al., 2025).

**Figure 3:**
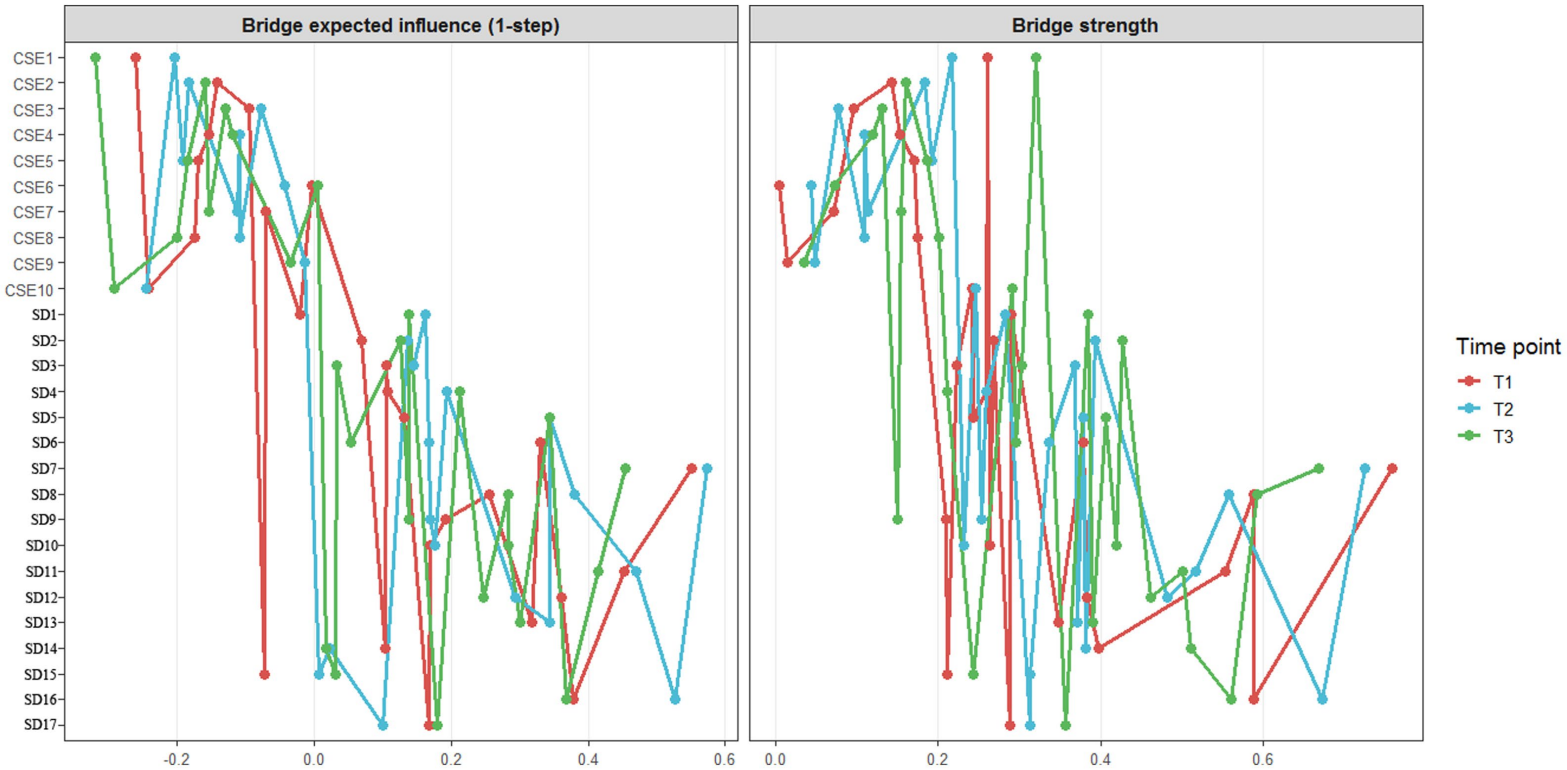
Bridge centrality plots for the nodes depicted as Bridge Strength, Bridge Expected Influence. The X-axis represents standardized z-scores of these three centrality indices (the higher the value, the more central the node), and the Y-axis represents the variables. Time1 = 2024.12, Time2 = 2025.4, Time3 = 2025.9, total sample = 896.

Bridge centrality indices were further calculated to identify nodes that connected the CSE and school disengagement (SD) communities within the network. As shown in Figure 3, CSE-related nodes generally exhibited relatively high bridge strength across the three time points. At T1, CSE1 (0.26), CSE10 (0.25), and CSE2 (0.19) showed the highest bridge strength values. At T2, CSE10 (0.26) remained the strongest bridge node, followed by CSE5 (0.20) and CSE1 (0.19). At T3, CSE1 (0.30) and CSE10 (0.28) continued to demonstrate relatively high bridge strength, while SD14 (0.27) also emerged as a prominent bridge node. These findings suggest that specific CSE items consistently showed relatively strong cross-community associations with school disengagement nodes across time points. In particular, items reflecting positive self-evaluation and perceptions of hopelessness appeared to play relatively important roles in linking the CSE and disengagement communities.

Regarding bridge expected influence, only relatively small values were observed at T3, with CSE6 (0.04) and SD9 (0.03) showing the highest positive bridge expected influence values. Overall, the bridge expected influence values were relatively modest, suggesting that cross-community associations were generally weaker than within-community associations in the present networks. Nevertheless, the relatively stable bridge strength patterns across time points indicate that several CSE nodes consistently maintained cross-dimensional connections with disengagement-related nodes. The detailed raw values for bridge centrality indices are presented in Table 4.

**Table 4:** List of nodes, the values of bridge centrality.

Variable	T1		T2		T3	
	Bridge strength	Bridge expected influence	Bridge strength	Bridge expected influence	Bridge strength	Bridge expected influence
CSE1	0.26	−0.26	0.19	−0.14	0.30	−0.30
CSE2	0.19	−0.12	0.17	−0.17	0.16	−0.16
CSE3	0.10	−0.10	0.07	−0.07	0.13	−0.13
CSE4	0.14	−0.14	0.15	−0.15	0.16	−0.16
CSE5	0.18	−0.18	0.20	−0.20	0.23	−0.19
CSE6	0.02	0.01	0.05	−0.04	0.15	0.03
CSE7	0.05	−0.05	0.11	−0.11	0.14	−0.14
CSE8	0.17	−0.17	0.12	−0.12	0.18	−0.18
CSE9	0.02	−0.02	0.08	0.00	0.05	−0.03
CSE10	0.25	−0.25	0.26	−0.26	0.28	−0.28
SD1	0.12	−0.12	0.04	−0.04	0.11	−0.11
SD2	0.09	−0.09	0.14	−0.14	0.12	−0.12
SD3	0.07	−0.07	0.09	−0.09	0.10	−0.10
SD4	0.07	−0.07	0.04	−0.04	0.01	−0.01
SD5	0.06	−0.06	0.04	−0.04	0.05	−0.05
SD6	0.08	−0.01	0.08	−0.08	0.15	−0.11
SD7	0.09	−0.09	0.08	−0.06	0.12	−0.10
SD8	0.18	−0.18	0.14	−0.08	0.17	−0.17
SD9	0.02	0.00	0.06	−0.04	0.05	0.03
SD10	0.06	−0.06	0.04	−0.00	0.06	−0.06
SD11	0.03	−0.03	0.02	−0.02	0.07	−0.07
SD12	0.01	−0.01	0.10	−0.10	0.12	−0.12
SD13	0.03	−0.03	0.01	−0.01	0.09	0.00
SD14	0.14	−0.14	0.18	−0.18	0.27	−0.24
SD15	0.15	−0.15	0.15	−0.15	0.10	−0.10
SD16	0.10	−0.10	0.08	−0.08	0.09	−0.09
SD17	0.06	−0.06	0.11	−0.11	0.10	−0.10

CSE, core self-evaluation; SD, school disengagement; Time1 = 2024.12, Time2 = 2025.4, Time3 = 2025.9, total sample = 896.

### 3.4 Network accuracy and stability

**Figure 4:**
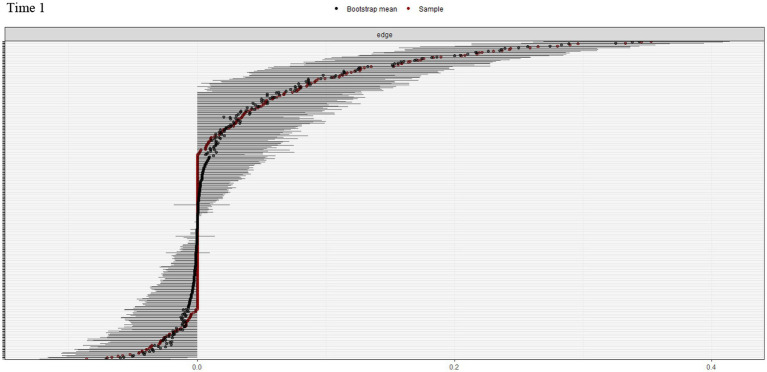
Nonparametric bootstrapped confidence intervals of estimated edges of all variables at T1 (2024.12). The red line represents the edge, as estimated in the sample. The grey indicates 95% bootstrapped confidence interval. The x-axis represents the edges, while specific edges are denoted along the y-axis by the grey lines.

**Figure 5:**
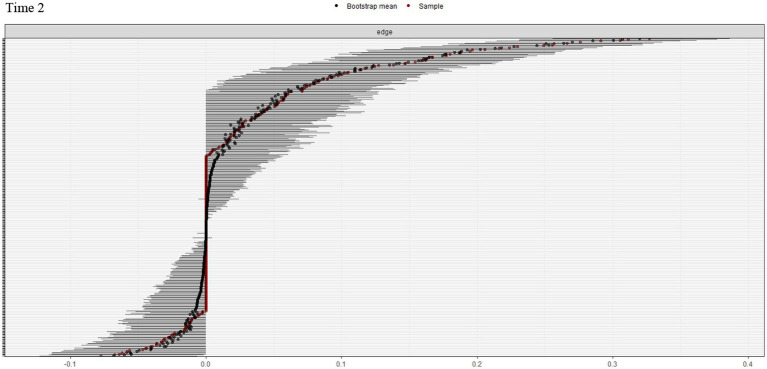
Nonparametric bootstrapped confidence intervals of estimated edges of all variables at T2 (2025.4). The red line represents the edge, as estimated in the sample. The grey indicates 95% bootstrapped confidence interval. The x-axis represents the edges, while specific edges are denoted along the y-axis by the grey lines.

**Figure 6:**
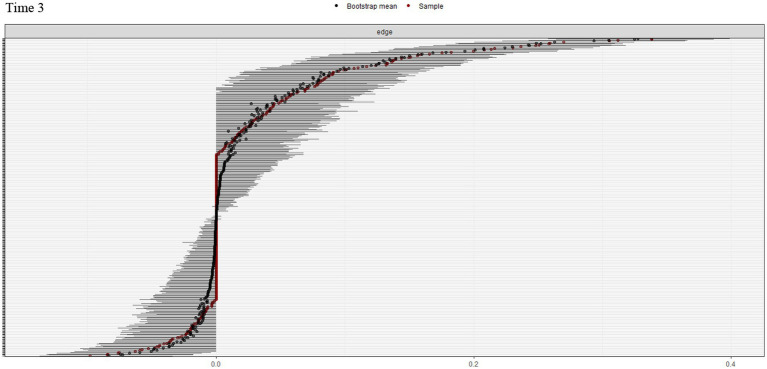
Nonparametric bootstrapped confidence intervals of estimated edges of all variables at T3 (2025.9). The red line represents the edge, as estimated in the sample. The grey indicates 95% bootstrapped confidence interval. The x-axis represents the edges, while specific edges are denoted along the y-axis by the grey lines.

The accuracy and stability of the network structures were assessed using bootstrap procedures. Figures 4–6 presents the 95% confidence intervals (CIs) of edge weights estimated from 1,000 bootstrap samples. Overall, the bootstrapped CIs were relatively narrow, indicating acceptable accuracy and precision of the estimated edge weights across the three time points. These findings suggest that the observed associations among core self-evaluations (CSE) and school disengagement (SD) nodes were estimated with acceptable accuracy.

**Figure 7:**
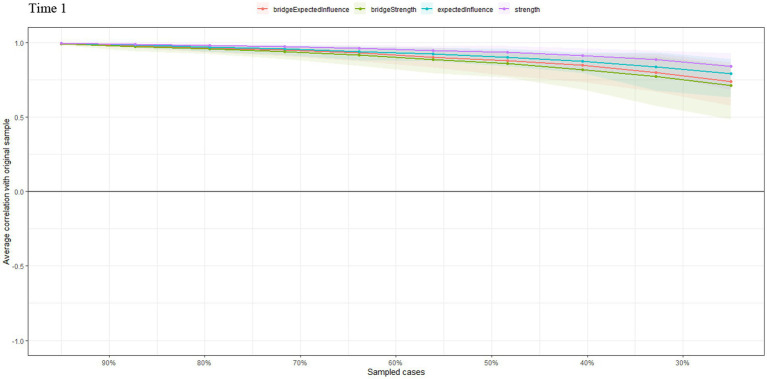
Stability estimations of centrality at T1 (2024.12) with the case-drop bootstrapping method.

**Figure 8:**
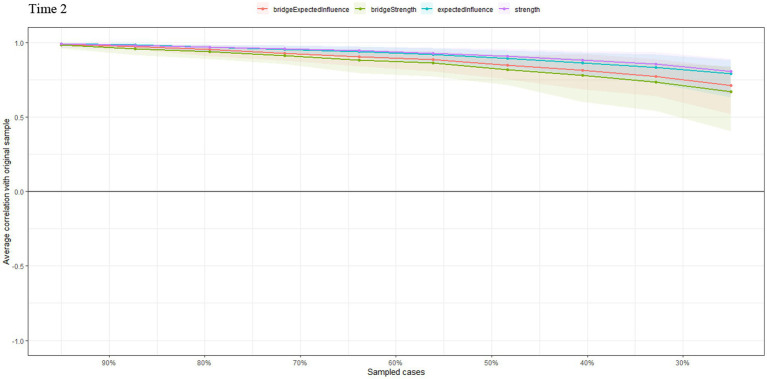
Stability estimations of centrality at T2 (2025.4) with the case-drop bootstrapping method.

**Figure 9:**
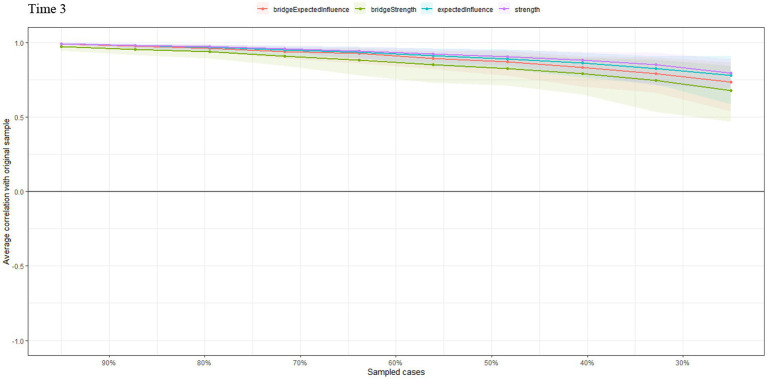
Stability estimations of centrality at T3 (2025.9) with the case-drop bootstrapping method.

Figures 7–9 demonstrates the stability of the centrality indices. The CS coefficients for strength, expected influence, bridge strength, and bridge expected influence were 0.75, 0.67, 0.60, and 0.67 at T1, respectively; 0.67, 0.67, 0.52, and 0.60 at T2, respectively; and 0.75, 0.67, 0.52, and 0.60 at T3, respectively. According to recommended criteria, CS coefficients above 0.50 indicate good stability of centrality indices (Epskamp et al., 2018). Therefore, the present results suggest that the estimated centrality and bridge centrality indices showed satisfactory stability across the three time points. In addition, Supplementary Figures S1–S6 present the bootstrap difference tests for node strength and expected influence at T1, T2, and T3, respectively, providing further information regarding differences between node centrality estimates across the networks.

### 3.5 Network comparison

Network Comparison Tests (NCTs) were conducted to examine differences in network structure and global strength across T1, T2, and T3. Holm correction was applied for multiple comparisons. The results of the structural invariance tests indicated that no significant differences in overall network structure were observed between T1 and T2 (M = 0.14, *p* = 0.25) or between T2 and T3 (M = 0.12, *p* = 0.39). Similarly, the comparison between T1 and T3 showed no statistically significant structural invariance difference after correction, although the result approached significance (M = 0.15, *p* = 0.06). These findings suggest that the overall configuration of associations among core self-evaluations (CSE) and school disengagement (SD) nodes remained relatively stable across the three time points.

For the global strength invariance tests, the reported *p*-values were unadjusted, consistent with the standard NCT procedure (van Borkulo et al., 2023). The global strength values for T1 and T2 were 12.47 and 12.36, respectively, with no significant difference observed between the two networks (S = 0.11, *p* = 0.49). Similarly, the comparison between T2 and T3 showed no significant difference in global strength (12.36 vs. 12.03; S = 0.33, *p* = 0.07), although the global strength value at T3 was numerically lower. In contrast, the comparison between T1 and T3 revealed a significant difference in global strength (12.47 vs. 12.03; S = 0.44, *p* = 0.02). Edge-level comparisons were also conducted; however, after Holm correction for multiple comparisons, no edge-level differences remained statistically significant in any of the three pairwise comparisons.

Overall, the NCT results suggest that the general network structure remained relatively consistent across time, while modest differences in overall connectivity emerged between T1 and T3. These findings provide quantitative evidence regarding longitudinal differences in the associations among CSE and school disengagement nodes while also indicating substantial structural consistency across the three measurement occasions.

## 4 Discussion

This study employed a three-wave longitudinal network analysis to examine structural associations between core self-evaluations (CSE) and school disengagement during early adolescence. By integrating cross-sectional network estimation with longitudinal comparisons, the present study provides a fine-grained perspective on how these constructs are interconnected over time. Overall, CSE was negatively associated with school disengagement across all three networks, whereas school disengagement showed relatively stronger within-community connectivity. Together, these findings suggest a relatively stable structural separation between self-evaluative resources and disengagement-related experiences within a network framework.

### 4.1 Cross-sectional structural associations

Across the three time points, CSE items generally showed negative cross-community associations with cognitive, behavioral, and emotional disengagement items. This pattern indicates that adolescents with more positive self-evaluations tend to report lower levels of disengagement-related cognitions, emotional exhaustion, and behavioral withdrawal, consistent with previous findings linking core self-evaluations to academic burnout-related outcomes (Ye et al., 2025). These associations were distributed across multiple item-level pathways rather than being confined to specific nodes, suggesting a broad structural linkage between self-evaluative resources and school disengagement within the network structure.

Within the disengagement network, cognitive disengagement consistently showed relatively high centrality across waves, suggesting that it occupied a relatively central position within the disengagement network. This finding is consistent with prior network-based evidence highlighting the importance of cognitive disengagement in academic burnout-related structures (Lin et al., 2025). Emotional disengagement showed increased centrality at T3, suggesting a greater integration with other disengagement components under sustained academic demands. Overall, these results highlight the interconnected nature of school disengagement and suggest that cognitive processes showed relatively strong associations with other disengagement components.

### 4.2 Longitudinal network comparison

Network comparison analyses indicated that the overall structure of associations between core self-evaluations (CSE) and school disengagement remained stable across the three time points, with no significant differences in structural invariance. This suggests that the configuration of relationships between self-evaluative resources and disengagement components is relatively consistent during early adolescence. However, differences in global network strength were observed between T1 and T3, indicating reduced overall connectivity in later waves. This pattern suggests that the strength of associations among psychological components differed across measurement occasions.

At the node level, cognitive disengagement consistently showed relatively high centrality across all waves, whereas emotional disengagement became more central at later time points. Negative cross-community associations between CSE and school disengagement remained stable over time, although their magnitude varied slightly. Overall, these findings suggest that while the structural organization of the network is largely stable, the strength of specific associations may fluctuate over time. These results are generally consistent with Conservation of Resources Theory, which emphasizing the associations between psychological resources and stress-related experiences (Hobfoll, 1989).

### 4.3 Theoretical and practical implications

The present study provides several theoretical implications. The findings extend variable-centered approaches by demonstrating that core self-evaluations and school disengagement are interconnected through multiple item-level pathways within a psychological network (Borsboom, 2017; Epskamp et al., 2018). Rather than functioning as independent constructs, they appear as a set of closely associated cognitive, emotional, and self-evaluative components. In particular, the central role of cognitive disengagement suggests that maladaptive learning cognitions showed relatively high centrality within the disengagement network, consistent with previous studies reporting close associations between maladaptive learning cognitions and disengagement-related experiences. From the perspective of Conservation of Resources Theory, core self-evaluations may be viewed as a relatively stable psychological characteristic associated with adolescents’ interpretations of and responses to academic stress (Hobfoll, 1989).

Practically, these findings suggest that interventions aimed at reducing school disengagement may benefit from targeting cognitive disengagement, particularly students’ perceptions of meaning and value in learning. Strengthening core self-evaluative resources such as self-efficacy, emotional stability, and perceived control may also relate to reduced disengagement-related experiences. Overall, the network perspective further implies that effective interventions should address school disengagement as a multidimensional construct characterized by interconnected components rather than focusing on isolated behavioral components (Borsboom, 2017; Robinaugh et al., 2016).

## 5 Limitation

Despite the insights provided by the three-wave longitudinal network analysis on core self-evaluations (CSE) and school disengagement, several limitations should be noted. First, regarding variable selection, the present study focused on CSE and school disengagement, while other relevant psychological and contextual factors, such as learning meaning, coping strategies, psychological resilience, parental stress, and peer pressure, were not included in the network model (Brassai et al., 2011; Zhou et al., 2023; Choi et al., 2023; Chockalingam et al., 2023; Devenney and O’Toole, 2021; Crumly et al., 2022). Therefore, the current findings reflect only a partial representation of adolescents’ learning-related psychological system, and future studies may expand the model by incorporating additional variables (Leduc et al., 2024).

Second, all variables were assessed using self-report measures, which may be influenced by social desirability and recall bias. Such limitations may also affect the estimation of network structures due to shared method variance and measurement error sensitivity (Podsakoff et al., 2003; de Ron et al., 2022). Future research could improve robustness by integrating multiple data sources. Third, although the longitudinal design allows examination of changes across time, the observed associations remain correlational and do not allow inferences about temporal ordering or causality. Future studies may address this issue using intensive longitudinal designs, such as experience sampling methods (Bringmann et al., 2015; Seçer et al., 2025). Finally, the sample was drawn from three junior high schools in one region of China, which may limit generalizability. Replication in other cultural and educational contexts is needed to strengthen external validity (Leduc et al., 2024; Bayly and Vasilenko, 2021).

## 6 Conclusion

This study employed a three-wave longitudinal network analysis to examine the associations between core self-evaluations (CSE) and school disengagement during early adolescence. Results showed a generally stable pattern of negative associations between CSE and school disengagement across the three time points, alongside stronger within-community connectivity among disengagement components. At the item level, cognitive disengagement consistently emerged as a relatively central node across the networks, while emotional disengagement showed increased centrality at later waves, suggesting some temporal variation in the internal organization of disengagement. Overall, the network structure remained largely stable over time, with only modest fluctuations in global connectivity and node centrality. These findings offer a fine-grained view of how self-evaluative resources and disengagement-related experiences are associated within a relatively stable psychological network structure in early adolescence, and suggest that interventions may benefit from focusing on central cognitive disengagement components while supporting adolescents’ core self-evaluative resources.

## Data Availability

The datasets presented in this study can be found in online repositories. The names of the repository/repositories and accession number(s) can be found below: https://osf.io/2vfz5/.
