# Supplementary material for: Core self-evaluation and school disengagement in early adolescence: a three-wave longitudinal network analysis
Source: Front Psychol. 2026 Jul 3;17:1809883. doi: 10.3389/fpsyg.2026.1809883 (PMC13382616; doi:10.3389/fpsyg.2026.1809883)
Supplement: Supplementary file 1 [file Supplementary_file_1.docx]

**Supplementary information**

**Table S1.** Fit Indices for Longitudinal Measurement Invariance Models

**Table S2.** Value of edge weight between variable.

**Table S3.** List of nodes, the values of centrality.

**Figure S1.** Nonparametric Bootstrapped difference test for node Strength at T1 (2024.12).

**Figure S2.** Nonparametric Bootstrapped difference test for node Strength at T2 (2025.4).

**Figure S3.** Nonparametric Bootstrapped difference test for node Strength at T3 (2025.9).

**Figure S4.** Nonparametric Bootstrapped difference test for node Expected Influence at T1 (2024.12).

**Figure S5.** Nonparametric Bootstrapped difference test for node Expected Influence at T2 (2025.4).

**Figure S6.** Nonparametric Bootstrapped difference test for node Expected Influence at T3 (2025.9).

**Table S1.** Fit Indices for Longitudinal Measurement Invariance Models

| Model | χ² | df | CFI | TLI | RMSEA | SRMR | ΔCFI | ΔRMSEA | Δχ² | Δdf |
| --- | --- | --- | --- | --- | --- | --- | --- | --- | --- | --- |
| configural | 1536.98 | 348 | 0.93 | 0.92 | 0.06 | 0.05 |  |  |  |  |
| metric | 1616.49 | 376 | 0.93 | 0.92 | 0.06 | 0.05 |  |  |  |  |
| scalar | 1718.74 | 404 | 0.93 | 0.92 | 0.06 | 0.05 |  |  |  |  |
|  |  |  |  |  |  |  |  |  |  |  |
| Comparison |  |  |  |  |  |  |  |  |  |  |
| Metric vs Configural | |  |  |  |  |  | -0.003 | -0.001 | 79.52 | 28 |
| Scalar vs Metric | |  |  |  |  |  | -0.004 | 0 | 102.25 | 28 |

**Table S2.** Value of edge weight between each variable.

| Node Pair | T1 | Node Pair | T2 | Node Pair | T3 |
| --- | --- | --- | --- | --- | --- |
| SD9 - SD10 | 0.37 | SD9 - SD10 | 0.34 | SD9 - SD10 | 0.35 |
| SD14 - SD15 | 0.36 | CSE2 - CSE3 | 0.33 | CSE2 - CSE3 | 0.35 |
| CSE2 - CSE3 | 0.35 | SD7 - SD8 | 0.32 | SD1 - SD2 | 0.32 |
| CSE7 - CSE8 | 0.30 | SD14 - SD15 | 0.31 | SD3 - SD4 | 0.32 |
| SD7 - SD8 | 0.29 | SD1 - SD3 | 0.28 | SD16 - SD17 | 0.28 |
| SD16 - SD17 | 0.28 | CSE1 - CSE4 | 0.27 | SD14 - SD15 | 0.27 |
| SD1 - SD2 | 0.28 | SD1 - SD2 | 0.27 | SD7 - SD8 | 0.27 |
| SD3 - SD4 | 0.27 | SD15 - SD17 | 0.27 | CSE5 - CSE8 | 0.26 |
| SD8 - SD9 | 0.27 | CSE4 - CSE6 | 0.27 | SD15 - SD17 | 0.26 |
| CSE1 - CSE6 | 0.26 | SD3 - SD4 | 0.24 | SD1 - SD3 | 0.24 |
| SD1 - SD3 | 0.24 | CSE1 - CSE6 | 0.24 | CSE7 - CSE8 | 0.23 |
| SD15 - SD17 | 0.23 | CSE7 - CSE8 | 0.22 | CSE4 - CSE9 | 0.22 |
| SD11 - SD12 | 0.23 | SD14 - SD17 | 0.22 | CSE1 - CSE4 | 0.22 |
| CSE4 - CSE9 | 0.22 | SD16 - SD17 | 0.21 | CSE4 - CSE6 | 0.21 |
| CSE5 - CSE7  CSE1 - CSE4  SD5 - SD6  SD15 - SD16  SD2 - SD3  CSE1 - CSE9  CSE7 - CSE10  CSE2 - CSE10  SD2 – SD4  CSE4 - CSE6  CSE8 - CSE10  CSE5 - CSE8  SD14 - SD17  SD14 - SD16  SD12 - SD13  SD6 - SD11  SD6 - SD12  CSE3 - CSE10  CSE3 - CSE7  CSE3 - CSE5  SD3 - SD5  SD5 - SD11  SD11 - SD13  SD13 - SD16  SD4 - SD17  SD4 - SD6  SD9 - SD13  SD6 - SD8  SD8 - SD10  SD7 – SD9  SD2 - SD6  SD2 - SD11  SD2 – SD5  SD1 – SD4  SD10 - SD15 | 0.22  0.21  0.21  0.20  0.19  0.18  0.18  0.18  0.17  0.17  0.17  0.16  0.16  0.14  0.14  0.14  0.14  0.14  0.13  0.13  0.12  0.12  0.11  0.11  0.11  0.10  0.10  0.10  0.10  0.09  0.09  0.09  0.09  0.09  0.08 | SD11 - SD12  CSE4 - CSE9  CSE6 - CSE9  CSE8 - CSE10  SD2 - SD3  CSE3 - CSE7  CSE7 - CSE10  SD2 - SD4  CSE5 - CSE8  SD1 - SD4  CSE2 - CSE10  SD8 - SD9  SD5 - SD6  SD6 - SD11  CSE1 - CSE9  SD14 - SD16  SD12 - SD13  CSE5 - CSE7  SD8 - SD10  SD7 - SD9  SD10 - SD13  CSE3 - CSE10  SD4 - SD6  SD6 - SD7  SD5 - SD7  SD4 - SD16  SD5 - SD16 | 0.20  0.20  0.20  0.19  0.17  0.17  0.17  0.17  0.16  0.16  0.16  0.16  0.15  0.15  0.15  0.15  0.13  0.13  0.12  0.12  0.12  0.11  0.11  0.11  0.11  0.10  0.10 | CSE1 - SD2 | -0.22 |
| CSE5 - SD8 | -0.21 |  |  |  |  |

**Table S3.** List of nodes, the values of centrality. ( z-scores)

| Variable | T1 | |  | T2 | |  | T3 | |
| --- | --- | --- | --- | --- | --- | --- | --- | --- |
|  | Strength | ExpectedInfluence |  | Strength | ExpectedInfluence |  | Strength | ExpectedInfluence |
| CSE1 | 0.70 | -1.32 |  | -0.22 | -1.01 |  | -0.03 | -2.14 |
| CSE2 | -0.21 | -0.81 |  | -0.05 | -1.22 |  | -0.27 | -0.69 |
| CSE3 | -0.41 | -0.65 |  | 0.06 | 0.40 |  | 0.12 | 0.06 |
| CSE4 | -1.26 | -1.52 |  | -0.58 | -1.05 |  | -0.80 | -1.06 |
| CSE5 | -0.88 | -1.78 |  | -0.91 | -1.96 |  | -0.07 | -1.11 |
| CSE6 | -1.83 | -0.41 |  | -0.46 | 0.32 |  | -0.49 | 0.41 |
| CSE7 | 0.69 | 1.21 |  | -0.17 | -0.40 |  | -0.22 | -0.33 |
| CSE8 | -0.86 | -1.68 |  | -0.65 | -0.71 |  | 0.19 | -0.74 |
| CSE9 | -2.72 | -1.73 |  | -2.64 | -1.57 |  | -2.78 | -1.25 |
| CSE10 | 1.12 | -0.84 |  | 1.15 | -1.19 |  | 0.98 | -1.12 |
| SD1 | 0.59 | 0.24 |  | 0.98 | 1.55 |  | 1.24 | 1.06 |
| SD2 | 0.91 | 0.86 |  | 1.65 | 0.77 |  | 2.68 | 2.08 |
| SD3 | 0.68 | 0.98 |  | 1.74 | 1.51 |  | 1.19 | 1.18 |
| SD4 | 0.28 | 0.64 |  | 0.73 | 1.34 |  | -0.36 | 1.06 |
| SD5 | -0.24 | 0.23 |  | -0.96 | 0.09 |  | 0.06 | 0.96 |
| SD6 | -0.32 | 0.40 |  | -1.05 | -0.51 |  | -1.09 | -0.89 |
| SD7 | 0.17 | 0.23 |  | 0.84 | 1.01 |  | -0.44 | -0.13 |
| SD8 | 1.19 | 0.01 |  | 1.09 | 0.69 |  | 0.96 | 0.14 |
| SD9 | -0.11 | 1.05 |  | -0.12 | 0.54 |  | -1.22 | 0.41 |
| SD10 | -0.06 | 0.46 |  | -0.60 | 0.53 |  | -0.05 | 0.73 |
| SD11 | 0.61 | 1.37 |  | 0.08 | 1.09 |  | -0.41 | 0.35 |
| SD12 | -0.73 | 0.48 |  | -0.88 | -0.63 |  | -0.64 | -0.50 |
| SD13 | -0.72 | 0.30 |  | -1.14 | 0.26 |  | -0.28 | 0.77 |
| SD14 | 0.76 | 0.16 |  | 0.50 | -0.60 |  | 0.33 | -1.32 |
| SD15 | 0.36 | -0.28 |  | -0.11 | -0.71 |  | -0.27 | 0.13 |
| SD16 | 2.05 | 1.73 |  | 1.35 | 1.35 |  | 0.73 | 0.95 |
| SD17 | 0.27 | 0.70 |  | 0.38 | 0.11 |  | 0.94 | 0.97 |

**
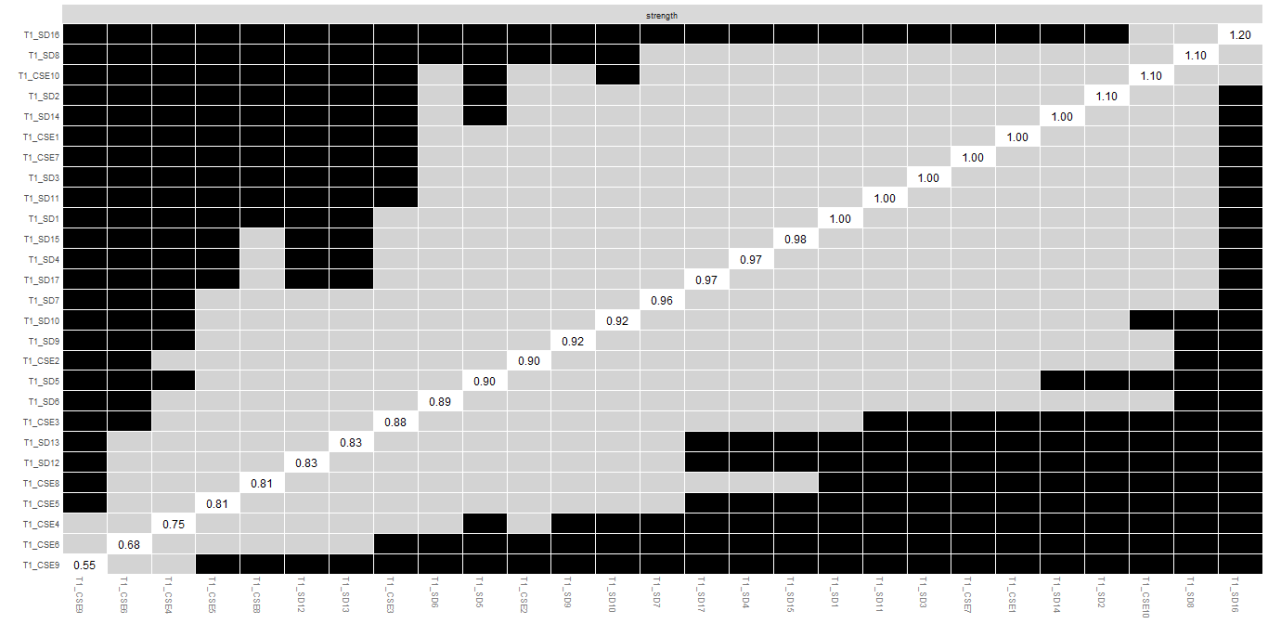
**

**Figure S1.** Nonparametric Bootstrapped difference test for node Strength at T1 (2024.12). Black boxes mean significant difference, while gray boxes mean no difference.

**
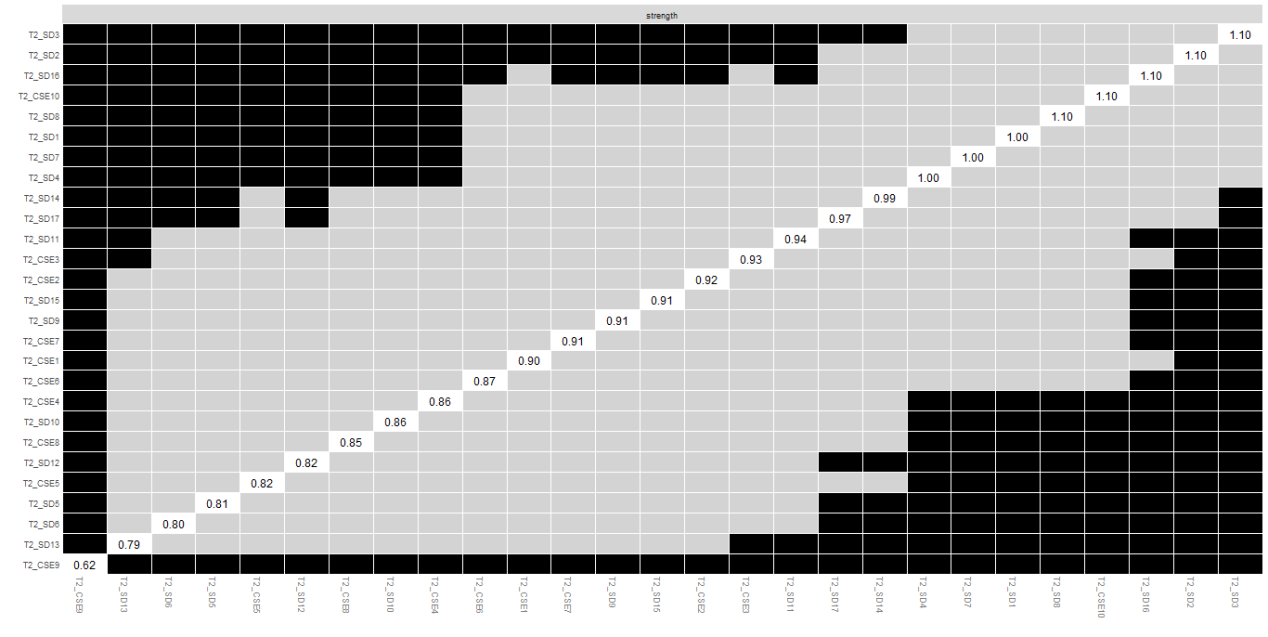
**

**Figure S2.** Nonparametric Bootstrapped difference test for node Strength at T2 (2025.4). Black boxes mean significant difference, while gray boxes mean no difference.

**
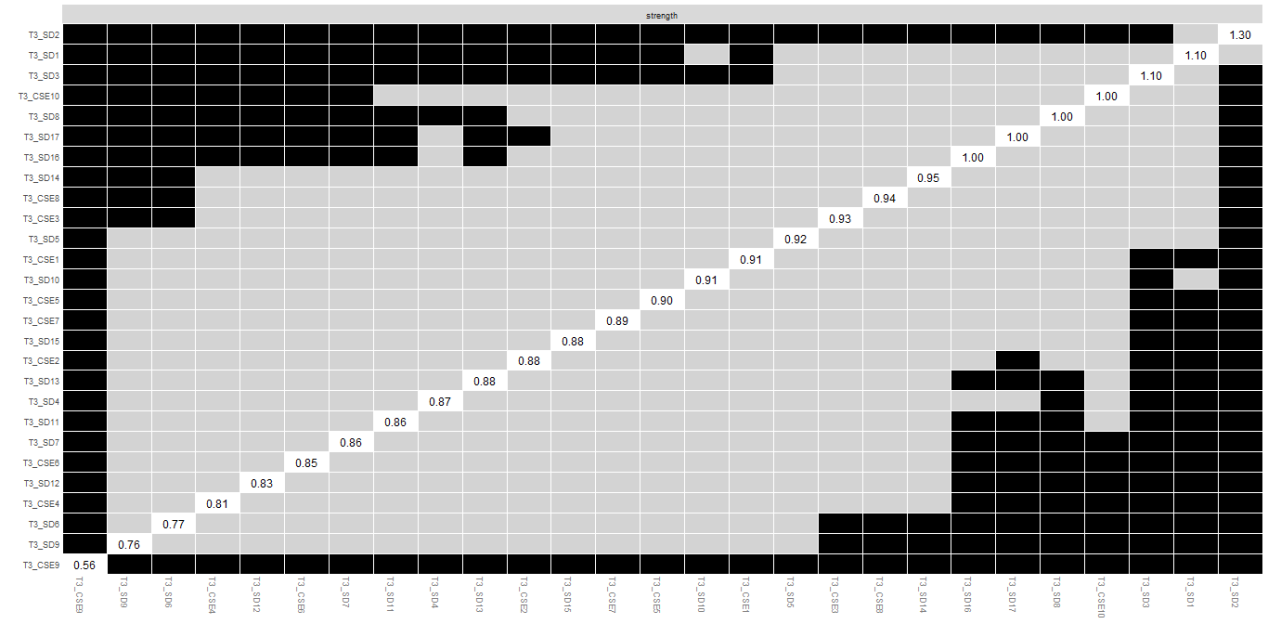
**

**Figure S3.** Nonparametric Bootstrapped difference test for node Strength at T3 (2025.9). Black boxes mean significant difference, while gray boxes mean no difference.

**
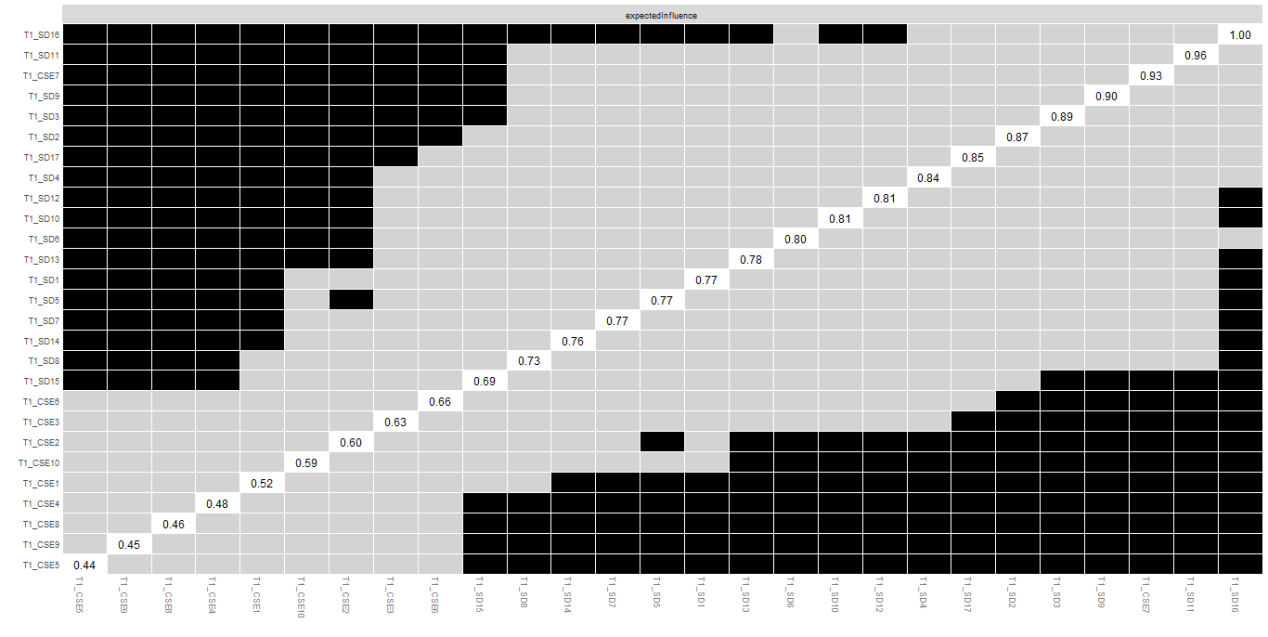
**

**Figure S4**. Nonparametric Bootstrapped difference test for node Expected Influence at T1 (2024.12). Black boxes mean significant difference, while gray boxes mean no difference.

**
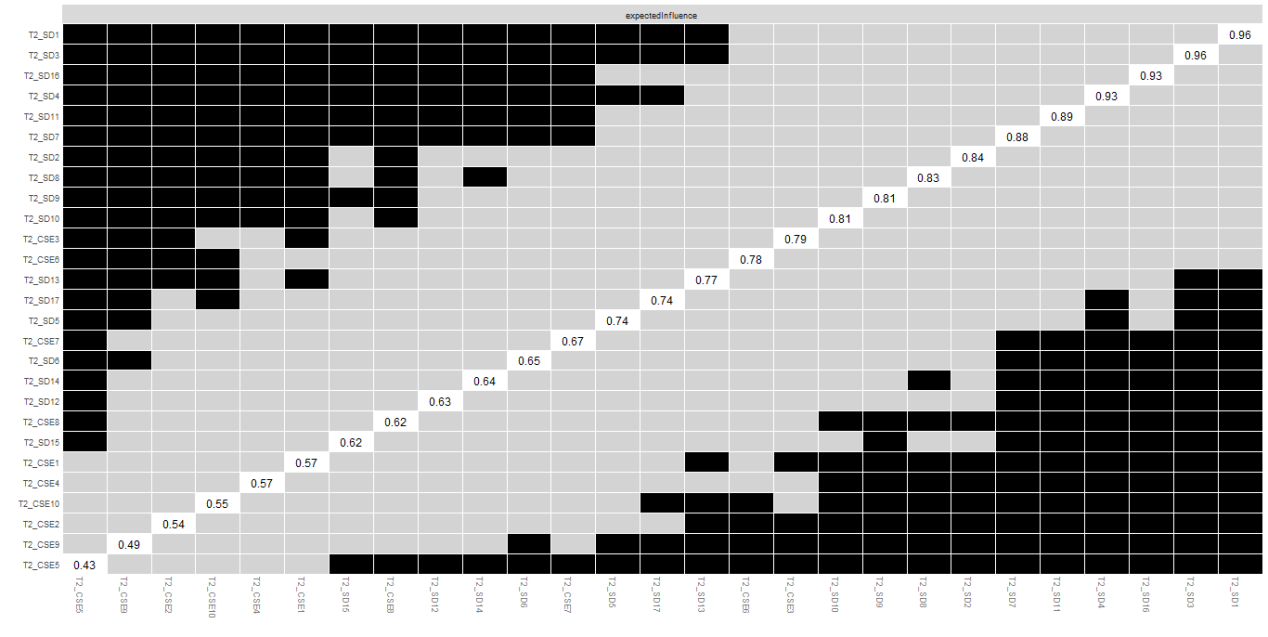
**

**Figure S5**. Nonparametric Bootstrapped difference test for node Expected Influence at T2 (2025.4). Black boxes mean significant difference, while gray boxes mean no difference.

**
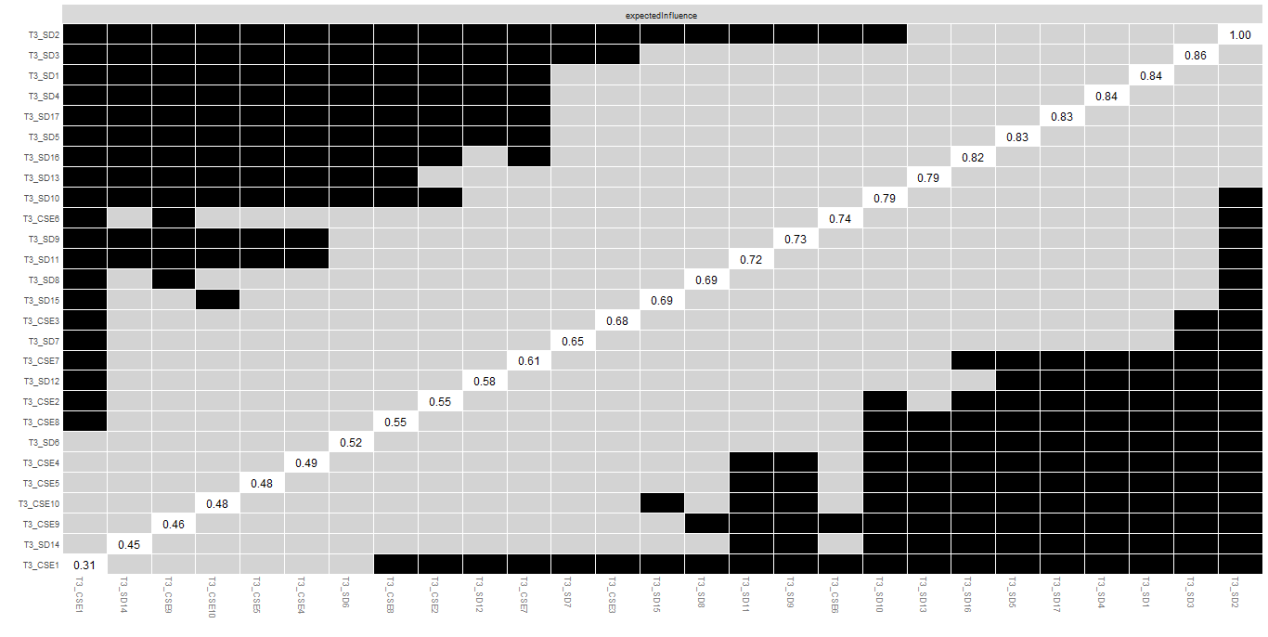
**

**Figure S6**. Nonparametric Bootstrapped difference test for node Expected Influence at T3 (2025.9). Black boxes mean significant difference, while gray boxes mean no difference.
