# Supplementary material for: Core self-evaluation and school disengagement in early adolescence: a three-wave longitudinal network analysis
Source: Front Psychol. 2026 Jul 3;17:1809883. doi: 10.3389/fpsyg.2026.1809883 (PMC13382616; doi:10.3389/fpsyg.2026.1809883)
Supplement: Supplementary file 2 [file Supplementary_file_2.docx]

**Appendix**

# Core Self-Evaluations Scale (CSE)

| Items | Strongly disagree | Disagree | Neutral | Agree | Strongly agree |
| --- | --- | --- | --- | --- | --- |
| CSE1. I believe I can achieve success in life. | 1 | 2 | 3 | 4 | 5 |
| CSE2. I often feel depressed. (R) | 1 | 2 | 3 | 4 | 5 |
| CSE3. When I fail, I feel worthless. (R) | 1 | 2 | 3 | 4 | 5 |
| CSE4. I can successfully complete various tasks. | 1 | 2 | 3 | 4 | 5 |
| CSE5. I feel uncertain about my studies. (R) | 1 | 2 | 3 | 4 | 5 |
| CSE6. Overall, I am satisfied with myself. | 1 | 2 | 3 | 4 | 5 |
| CSE7. I doubt my own abilities. (R) | 1 | 2 | 3 | 4 | 5 |
| CSE8. I feel uncertain about achieving success in my career. (R) | 1 | 2 | 3 | 4 | 5 |
| CSE9. I am capable of handling most of my problems. | 1 | 2 | 3 | 4 | 5 |
| CSE10. Many things seem hopeless to me. (R) | 1 | 2 | 3 | 4 | 5 |

**Note.** Items marked with “R” were reverse-coded prior to analysis (1=5, 2=4, 3=3, 4=2, 5=1).

1. Junior High School Students' School Disengagement Scale (SD)

| Items | Strongly disagree | Disagree | Neutral | Agree | Strongly agree |
| --- | --- | --- | --- | --- | --- |
| SD1. I think studying or learning has no meaning at all. | 1 | 2 | 3 | 4 | 5 |
| SD2. I only study because of pressure from parents or teachers. | 1 | 2 | 3 | 4 | 5 |
| SD3. I get no enjoyment from studying or learning. | 1 | 2 | 3 | 4 | 5 |
| SD4. I consider studying or learning to be a burden. | 1 | 2 | 3 | 4 | 5 |
| SD5. I feel that my days at school are just passing time. | 1 | 2 | 3 | 4 | 5 |
| SD6. I feel that much of what the teachers teach is useless to me. | 1 | 2 | 3 | 4 | 5 |
| SD7. I often lose concentration in class and easily get distracted. | 1 | 2 | 3 | 4 | 5 |
| SD8. My self-control in studying or learning is very poor. | 1 | 2 | 3 | 4 | 5 |
| SD9. I do not have the habit of previewing lessons before class or reviewing after class. | 1 | 2 | 3 | 4 | 5 |
| SD10. I do not make study plans based on learning tasks. | 1 | 2 | 3 | 4 | 5 |
| SD11. I often try every means to avoid studying or learning. | 1 | 2 | 3 | 4 | 5 |
| SD12. I often cannot complete homework independently. | 1 | 2 | 3 | 4 | 5 |
| SD13. I constantly think about going online to play games, chat, watch videos, etc. | 1 | 2 | 3 | 4 | 5 |
| SD14. Studying or learning often makes me feel nervous and anxious inside. | 1 | 2 | 3 | 4 | 5 |
| SD15. Studying/learning often makes me feel pressured. | 1 | 2 | 3 | 4 | 5 |
| SD16. As soon as I start studying or learning, I feel irritated and restless. | 1 | 2 | 3 | 4 | 5 |
| SD17. Studying or learning often makes me feel physically and mentally exhausted. | 1 | 2 | 3 | 4 | 5 |
